# Unawareness of deficits in mild cognitive impairment: a systematic review of its role in progression to Alzheimer’s disease

**DOI:** 10.1186/s12883-026-04884-8

**Published:** 2026-05-20

**Authors:** Lauren Moore, Astrid E. Lund, Charlotte Russell

**Affiliations:** Department of Psychology, Institute of Psychiatry, Psychology & Neuroscience, King’s College London, Addison House, Guy’s Campus, London, SE1 1UL UK

**Keywords:** Anosagnosia, Awareness, Mild Cognitive Impairment, Alzheimer’s Disease

## Abstract

**Background:**

Reduced awareness or poor insight into cognitive abilities is a well-documented feature of Alzheimer’s disease, yet its role in the earlier stages of cognitive decline—particularly in individuals with mild cognitive impairment (MCI)—remains less clear. Understanding whether diminished awareness in MCI is a predictor of progression to dementia is crucial, as it may help identify individuals who are at greater risk and who could benefit from timely support and intervention. This systematic review evaluates the evidence linking reduced awareness in MCI with an increased likelihood of conversion to dementia.

**Method:**

Four electronic databases (CINAHL, Medline, Embase and PsychInfo) were systematically searched for all studies assessing awareness in individuals with MCI, which tracked their cognitive status over time. The protocol was registered with PROSPERO and PRISMA guidelines were followed. Inclusion criteria—studies must: Include participants with confirmed MCI diagnosis; Assess the relationship between awareness of cognitive and/or functional abilities and the development of dementia; Have longitudinal design; Be peer-reviewed. Exclusion criteria—studies must not: Be published in a different language to English; Include participants with comorbid neurological conditions; Include participants from the same cohort as another study; Use a case series design.

Eleven studies were identified as fulfilling all criteria. Study quality was evaluated using the Critical Appraisal Skills Programme (CASP) checklist for cohort studies.

**Results:**

Six studies reported a statistically significant association between reduced awareness and conversion to dementia. Four studies found a trend toward significance, suggesting a possible link, but either did not test for significance or failed to reach it. Only one study found no association. Study quality was rated as high in five studies, moderate in two, and low in four. Notably, higher-quality studies were more likely to report significant associations. Due to substantial methodological variability across studies, a meta-analysis was not feasible.

**Conclusions:**

Reduced awareness of memory impairment appears predictive of increased risk of progression from MCI to dementia. Assessing awareness—through informant reports and/or comparisons between subjective and objective cognitive measures—could help identify individuals at elevated risk. These individuals may benefit from closer monitoring to facilitate timely diagnosis and intervention.

**Supplementary Information:**

The online version contains supplementary material available at 10.1186/s12883-026-04884-8.

## Background

Mild Cognitive Impairment (MCI) involves cognitive decline beyond that expected in normal aging, without impairing daily functioning [1]. Global prevalence in those over 50 is estimated at 15.56% [2], though reported rates vary widely [3]. While some individuals diagnosed with MCI remain stable or revert to normal cognition, this diagnosis increases the risk of dementia [4–6]. Therefore, understanding which factors predict progression to dementia is critical. Although at present no cure exists, early diagnosis enables timely support and access to available treatments, which in many countries can now include disease modifying amyloid antibodies (Lecanemab and Donanemab) [7].

Research has identified vascular issues, neuropsychiatric symptoms, and poor initial cognitive scores as risk factors for dementia [8–13]. However, a core feature of dementia, especially Alzheimer’s Disease (AD), is reduced awareness of cognitive or functional decline [14, 15], raising the possibility that this lack of awareness might itself be considered a risk factor.

Investigation of the role of awareness in conversion from MCI to AD is impacted by both inconsistent use of terminology and the plethora of methods used to quantify its presence. Terms like anosagnosia, impaired insight, and unawareness are used interchangeably [16–19]; here, we will use “awareness” for consistency. There are multiple ways to quantify awareness, with no ‘gold standard’ [e.g., 20]. Three main types of method are:

1. Denial of Impairment: Patients are classified as unaware if they deny cognitive decline, assessed via simple questions or clinician judgements [21, 22].
2. Patient-Informant Discrepancy: Patients and informants (e.g., family) complete parallel questionnaires, with score differences indicating awareness levels of the patient [23, 24].
3. Subjective vs. Objective Cognition: Awareness is assessed by comparing predicted or perceived performance with actual results [25], often used in metacognition studies [26].

These methods themselves vary considerably, For example, informant reliability varies due to relationship dynamics and potential bias [27], but – importantly—these reports often correlate with patients’ objective performance, unlike the patients’ self-reports [28, 29]. The lack of consensus around whether impairments in awareness of cognition is a risk factor for development of dementia likely reflect these methodological differences [30, 31]. Given that reduced awareness is common in AD, any impairment in awareness in MCI might indicate a more advanced decline and predict dementia progression. Although this hypothesis has been proposed [20, 31], no systematic review has yet evaluated whether impaired awareness independently predicts MCI conversion to dementia.

### Review questions

The questions asked in this systematic review were:

1. Is lack of awareness of cognitive impairment an independent risk factor of the progression from MCI to dementia?
2. Does the predictive ability of awareness as a risk factor vary depending on how it is measured?

## Methods

### Search strategy

The protocol was registered with the International Prospective Register of Systematic Reviews (PROSPERO; protocol number: CRD42023391119) in February 2023. The review followed the Preferred Reporting Items for Systematic Reviews and Meta Analyses (PRISMA) guidelines [32]. A computer-based search was conducted using four electronic literature databases (Medline, Embase, PsycINFO and CINAHL) to check title/abstracts of identify peer-reviewed studies written in English. An initial search was conducted in June 2023, followed by a second search in November 2024. Search terms were generated by reviewing key words and Medical Subject Headings (MeSH) from relevant published papers. MeSH terms and free text search terms varied for each database but included (cognit* adj4 impair* OR MCI) AND (introspect* OR insight OR awareness OR anosognosia OR metacognit*) AND (predict* OR progress* OR conver* OR transition) AND (Dementia OR Alzheimer*) AND (longitudinal.tw. OR retrospective.tw. OR exp cohort studies/OR (follow up adj (study or studies)).tw.). The full search strategy is included in Supplementary Information (A). Search terms were reviewed by all authors and a librarian. Forward and backward citation searching was carried out for each study to identify relevant papers missed by the original search.

### Study selection

**Fig. 1:**
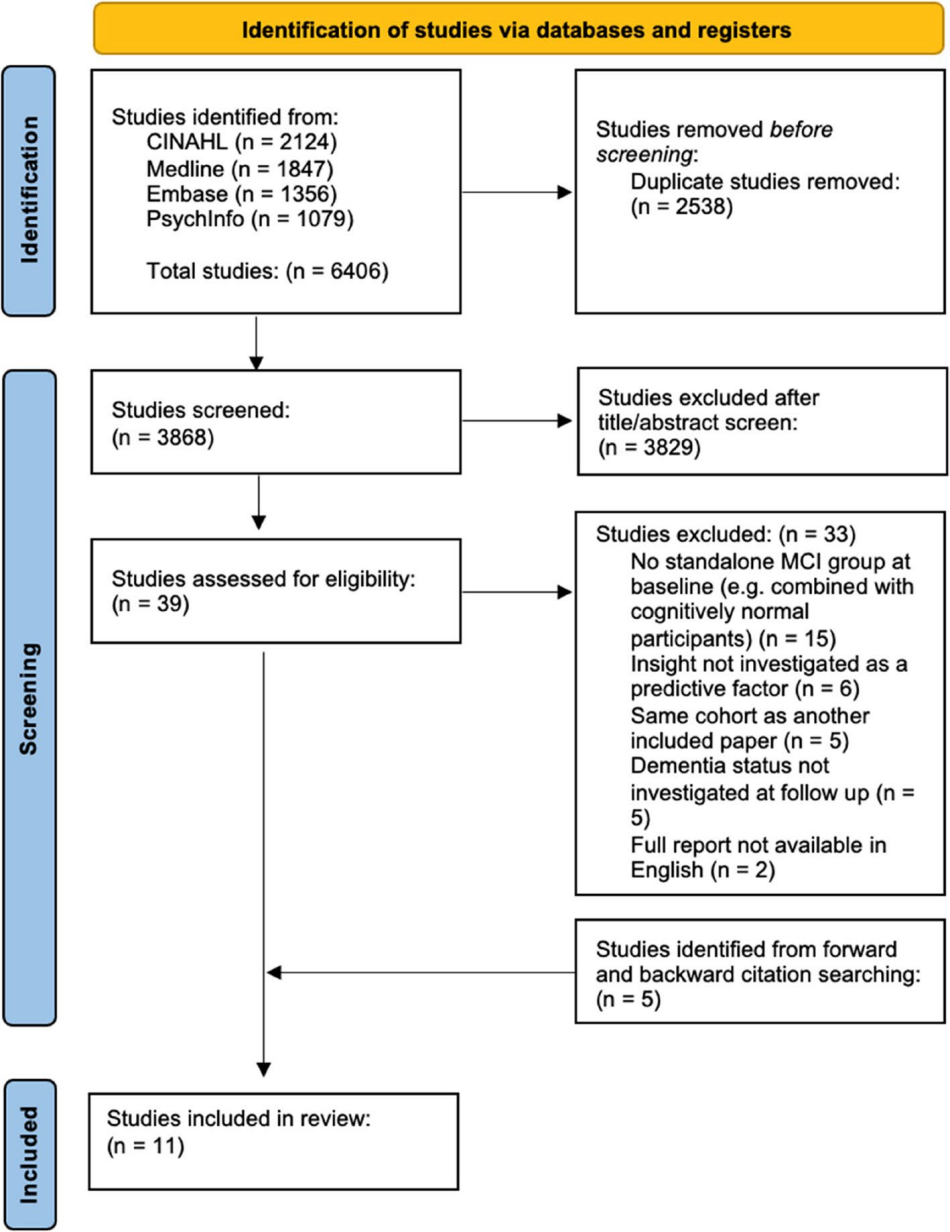
PRISMA flowchart of study selection

After removal of duplicates, 3868 titles and abstracts and subsequently 39 full text papers were screened for eligibility, leading to 11 papers included in the review (see Fig. 1).

Inclusion criteria; studies must:

- Include participants with a confirmed diagnosis of MCI.
- Assess the relationship between awareness of cognitive and/or functional abilities and the development of dementia.
- Have a longitudinal study design.
- Be peer-reviewed.

Exclusion criteria; studies must not:

- Be published in a different language to English.
- Include participants with comorbid neurological conditions.
- Include participants from the same cohort as another study included in the review.
- Use case study or case series design.

A second reviewer independently screened 20% of the articles at each stage of the search. The agreement rate was 99% and 71% respectively at each stage, and disagreements were successfully resolved through discussion.

### Data extraction

The following variables of each study were extracted for the Cochrane Data Extraction Form: author, year of publication, country, source of data, number recruited at baseline, number analysed, age, sex, years of education of participants (Table 1). Information on the definitions of key terms was also extracted: MCI diagnostic criteria, dementia diagnostic criteria, and how awareness was measured (Table 2). Finally, measures of association between awareness and the development of dementia were extracted: Dementia subtype; follow up duration; statistical methods; % conversion to dementia; reported association between awareness and dementia (Table 3). A second reviewer completed data extraction for a randomly selected 30% of the studies. Any disagreement was noted and resolved through discussion.

**Table 1:** Main methodological characteristics of each study

Reference	Country	Source of data	N recruited	N included in analysis	Age at baseline (years)	Sex M:F (%)	Mean years of education (SD)
Tabert et al*.,* 2002 [33]	USA	Memory clinic	107	92	67.6 ± 10.1	49:51	15.2 ± 3.6
Nobili et al*.,* 2010 [34]	Italy	Memory clinic	42	40	73.7 ± 7.5	43:57	’Aware’: 9.5 ± 4.6 ‘Unaware’: 9.5 ± 3
Gifford et al*.,* 2014 [22]	USA	NACC database	1843	1843	74.4 ± 7.5	48:52	15.6 ± 6.1
Spalletta et al*.,* 2014 [23]	Italy	Memory clinic	70	36	71 ± 5.9	53:47	Converters: 12.2 ± 6.2 Non-converters: 7.4 ± 3.2
Wolfsgruber et al*.,* 2014 [35]	Germany	German DCN	813	19	65.6 ± 7.93	59:41	12.6 ± 2.84
Silva et al*.,* 2016 [36]	Austria	Memory clinic	34	34	naMCI: 70 (55–79)* aMCI: 71 (57–85)*	50:50	8 (5–17)*
Cova et al*.,* 2017 [21]	Italy	Memory clinic	469	282	75.2 ± 6.7	44:56	7.7 ± 4
Gerretsen et al*.,* 2017 [24]	Canada	ADNI	525	499	Converters: 73.6 ± 7.5 Non-converters: 72.3 ± 7.8	Converters: 56:44 Non-converters: 57:43	Converters: 16 ± 2.7 Non-converters: 16.1 ± 2.7
Munro et al*.,* 2018 [37]	USA	Harvard Aging Brain Study	33	33	74.6 ± 8.5	73:27	16.7 ± 2.3
Bastin et al*.,* 2021 [25]	Belgium	Memory clinic	44	44	Converters: 75.3 ± 4.6 Non-converters: 72.3 ± 7.6	59:41	Converters: 13.2 ± 3.1 Non-converters: 12.6 ± 3.7
Chang et al*.,* 2023 [38]	USA	Einstein Aging Study	1097	676	78.5 ± 5.4	38:62	14 ± 3.5

*NACC* National Alzheimer’s Coordinating Center, *DCN* Dementia Competence Network, *naMCI* Non-amnestic MCI, *aMCI* Amnestic MCI, *ADNI* Alzheimer’s Disease Neuroimaging Initiative

^*^Range

**Table 2:**
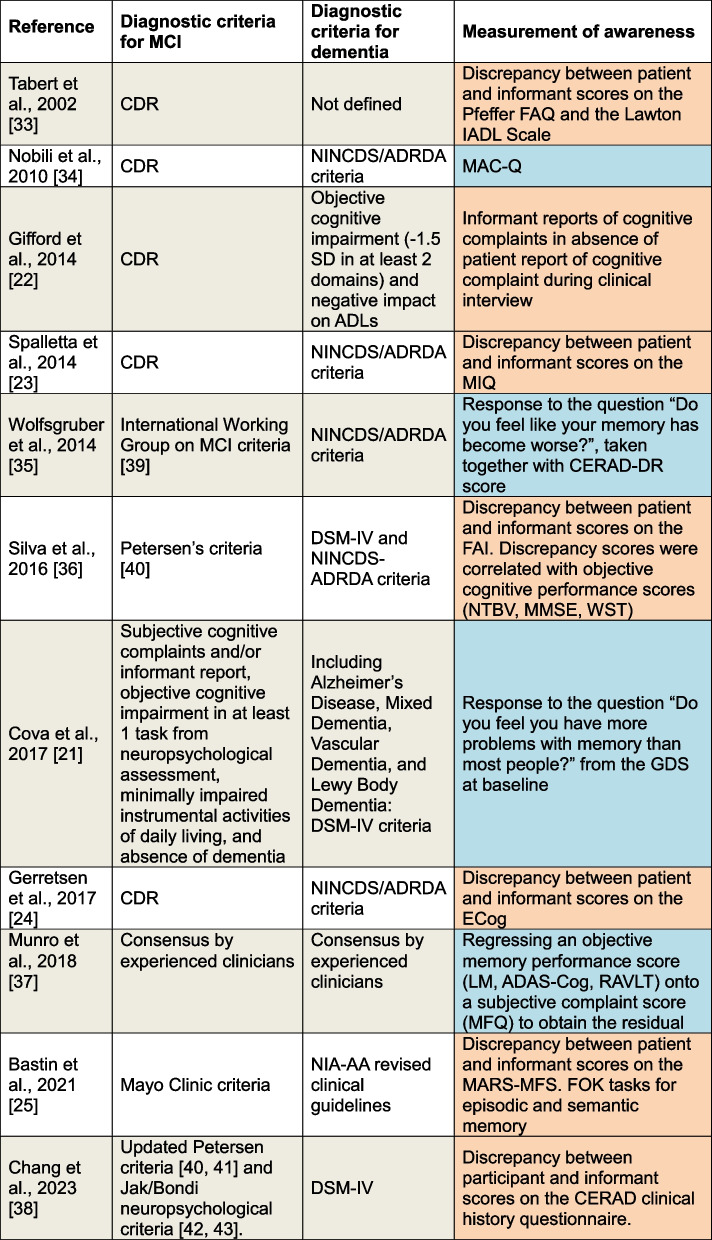
Criteria for MCI, dementia, and awareness used in each study [21–25, 33–43]

*Abbreviations*: *CDR* Clinical Dementia Rating Scale, *FAQ* Functional Activities Questionnaire, *IADL* Independent Activities of Daily Living, *NINCDS/ADRDA* National Institute of Neurological and Communicative Disorders and Stroke/Alzheimer’s Disease and Related Disorders Association, *MAC-Q* Memory Complaint Questionnaire, *MIQ* Memory Insight Questionnaire, *CERAD-DR* Consortium to Establish a Registry for Alzheimer’s Disease-Delayed Recall, *DSM-IV* Diagnostic and Statistical Manual for Mental Disorders-4th Edition, *FAI* Forgetfulness Assessment Inventory, *NTBV* Neuropsychology Test Battery Vienna, *MMSE* Mini Mental State Examination, *WST* The Wortschatztest, *GDS* Geriatric Depression Scale, *ECog-PR* Everyday Cognition – Partner Report, *ECog-SR* Everyday Cognition – Self Report, *LM* Logical Memory, *ADAS-Cog* Alzheimer’s Disease Assessment Scale – Cognitive subscale, *RAVLT* Rey Auditory Verbal Learning Test, *MFQ* Memory Functioning Questionnaire, *NIA-AA* National Institute on Aging and Alzheimer’s Association, *MARS-MFS* Memory Awareness Rating Scale – Memory Functioning Subscale, *FOK* Feeling of Knowing

Colour coding: ,

**Table 3:**
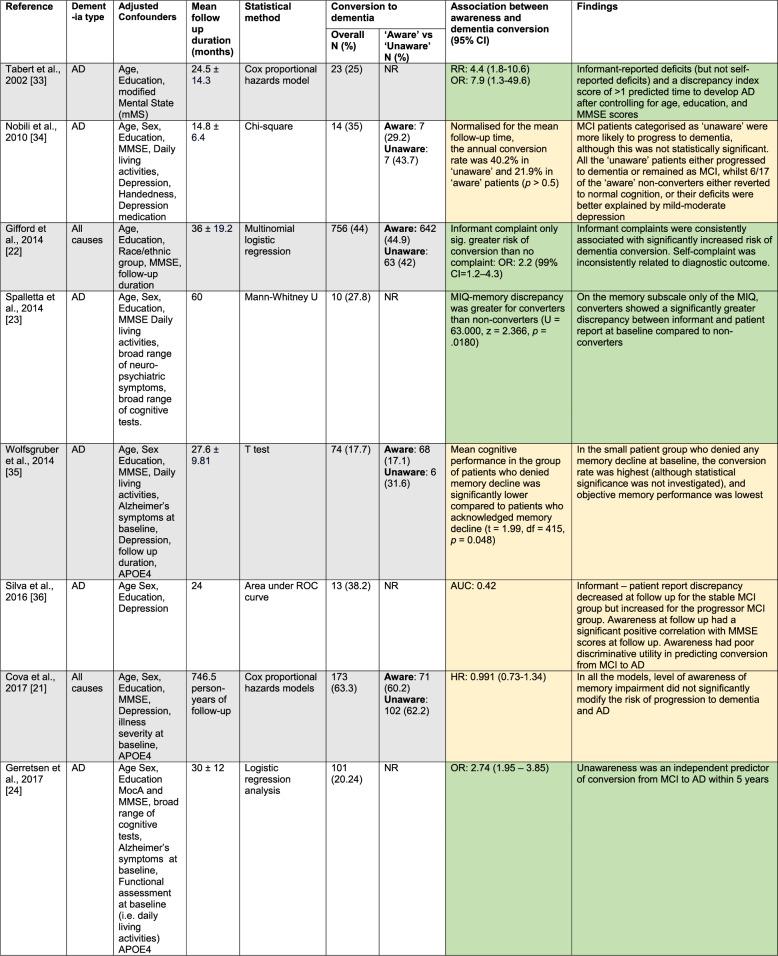

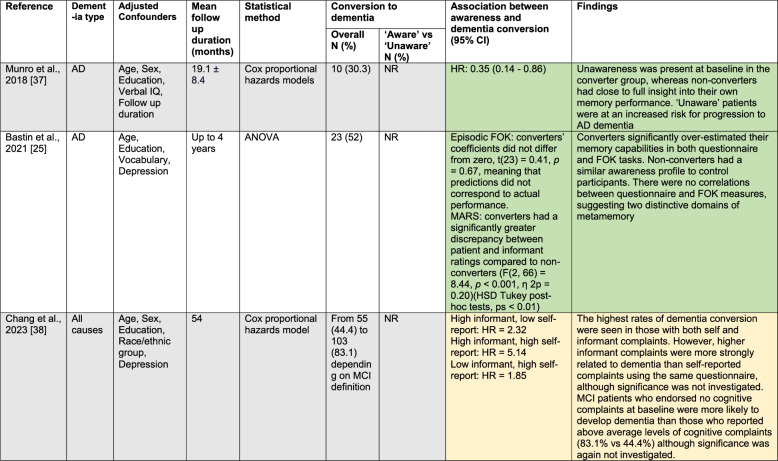
Relevant results and core methodological features from included papers. This includes the association between awareness and dementia. Colour coding in final columns denotes the association: green is evidence of significant association; yellow no significant or not statistically tested association [21–25, 33–38]

*AD* Alzheimer’s Disease, *MMSE* Mini Mental State Examination, *RR* Relative Risk, *OR* Odds Ratio, *NR* Not Reported, *ROC* Receiver-Operating-Characteristics, *AUC* Area under the ROC curve, *HR* Hazard Ratio

Colour coding:  between awareness and dementia,  between awareness and dementia

### Quality assessment

The quality of studies was assessed using the Critical Appraisal Skills Programme (CASP) checklist for cohort studies (see Table 4; CASP, 2018). The checklist begins with 2 screening items, followed by 10 additional items, all of the questions asked on the checklist for each paper can be seen at the top of Table 4. All 11 studies included in the review were scored on the 12 items by 2 reviewers—by rating ‘yes’, ‘no’ or ‘cannot tell’ for each. Studies were categorised as high, moderate, or low quality based on the proportion of yes/no/can’t tell ratings; this strategy is that used by other similar reviews [e.g., 44, 45, 46]. Disagreements between the reviewers were successfully resolved through discussion.

**Table 4:**
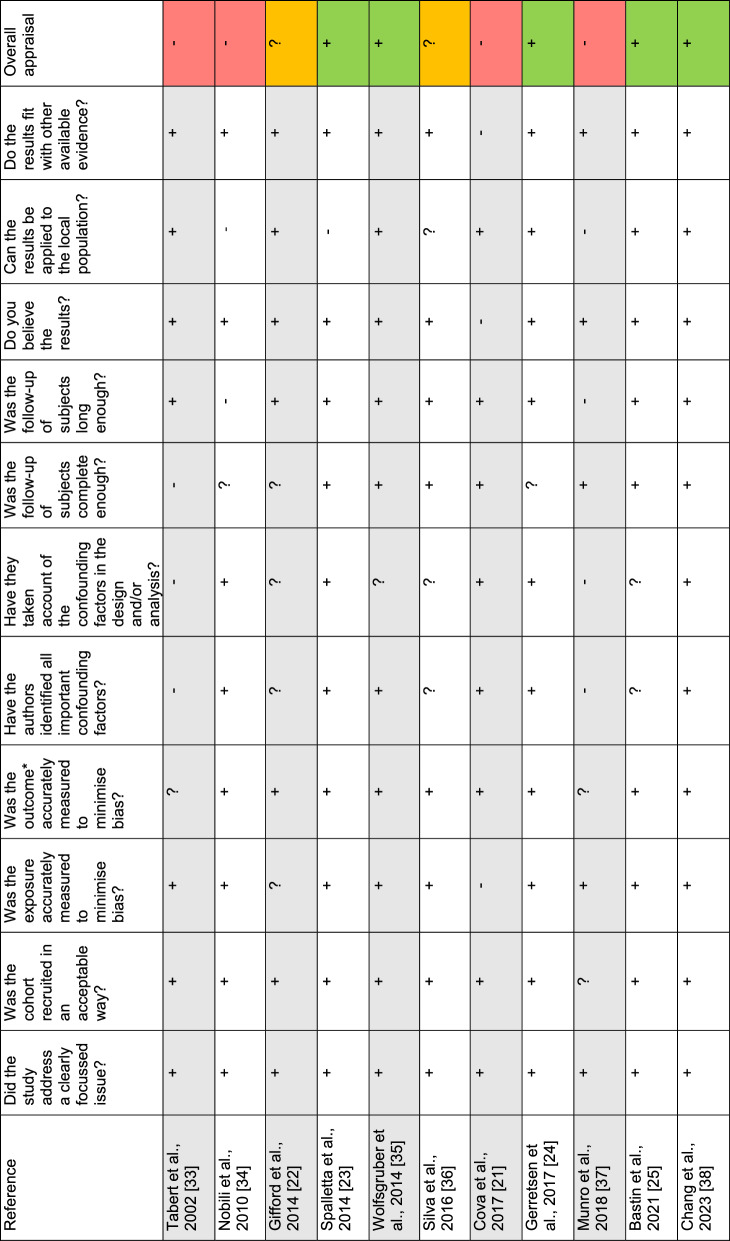
Assessment of quality of all included studies according to ratings from Critical Appraisal Skills Programme (CASP) checklists [21–25, 33–38]

Key: + Yes;—No;? Can’t tell

Exposure: measure of awareness

Outcome: dementia conversion

## Results

### Quality assessment

See Table 4 for the scores agreed by reviewers on this scale. The majority of studies (*N* = 10) passed the initial 2 screening questions of CASP. The exception was [27] where it was not possible to ascertain whether the cohort was recruited in an acceptable way, all other studies had stated their exact recruitment source – e.g., memory clinic or specific database. Overall appraisal was high for 5 studies [23–25, 35, 38] moderate for 2 studies [22, 36] and weak for 4 studies [21, 26, 33, 34].

All studies identified basic confounding variables such as age and years of education. Some studies accounted for additional confounding factors, such as baseline MMSE/MoCA scores [21, 22, 24], depressive symptoms [21, 35], APOE4 status [21, 24, 35], follow-up duration [22], physical comorbidities [21], ethnicity [38] and in medication which may impact cognition [34]. Some studies did not appear to account for possible confounding variables relating specifically to their study design, for example Bastin et al. (2021) did not take a measure of anxiety when looking at FOK tasks - anxiety has been shown to confound metacognitive performance, and Gifford et al. (2014) did not specify whether patients and informants were interviewed together or separately.

Five studies had a small sample size, limiting generalisability (Munro et al., 2018, *N* = 33; Silva et al., 2016, *N* = 34; Spalletta et al., 2014, *N* = 36; Nobili et al., 2010, *N* = 40; Bastin et al., 2021, *N* = 44). Only three studies [22, 23, 38] reported on ethnicity and not including this might impact the generalisability of findings. Two studies lacked a long enough follow up duration to draw meaningful conclusions (Nobili et al., 2010, mean duration: 14.8 months; Munro et al., 2018, mean duration: 19.1 months), which was acknowledged by the authors in their conclusions and as such was determined too short in the evaluation here. Studies which received a negative rating for follow-up were unclear about which participants were excluded vs lost to follow-up [33], whether there were any dropouts at all [22], or whether those who were lost to follow-up differed from the main cohort in any way. Some studies were also unclear about the depth of follow up and simply said “at least 1 follow up in 5 years” [24]. Some are ongoing studies so by definition are incomplete [34].

### Study characteristics

Duration of follow-up ranged from 14.8 months [32] to 60 months [14]. Six studies recruited directly from memory clinics whilst the other 5 used a larger network or database. All studies were published in peer reviewed journals from 5 different countries (USA (*n* = 4), Italy (*n* = 3), Austria (*n* = 1), Germany (*n* = 1), Canada (*n* = 1) and Belgium (*n* = 1) between 2002 and 2023. Seven studies recruited MCI patients only, 4 included a control group. Of those 4, 2 studies included healthy controls specifically matched for age, sex, and years of education [27, 33], the other 2 studies recruited cognitively normal controls through university programmes for older adults [34] and through the National Alzheimer’s Coordinating Center (NACC) database [22].

### Sample characteristics

Sample sizes ranged from 33 [37] to 1843 at baseline [22]. Mean age at baseline ranged from 65.6 years [35] to 78.5 [38]. Studies generally had an even split between males and females, except Munro et al. (2018) where 73% (*n* = 24) of the sample was male. Mean years of education ranged from 7.7 ± 4 [12] to 16.7 ± 2.3 [27]. Only 3 studies reported information about ethnicity. Gifford et al. (2014) reported their MCI cohort as 81% White and Chang et al. (2023) stated the subset of their cohort with an informant was 74% non-Hispanic White. Spalletta et al. (2014) reported their sample as homogenous regarding ethnicity. Six studies included patients with non-specific MCI diagnoses [22, 24, 28, 33, 36, 38] whilst 5 studies only recruited the amnestic subtype (aMCI). Three studies investigated all causes of dementia at follow-up [21, 22, 37] while 8 studies limited follow-up to Alzheimer’s Disease. Note this may not be clear from Table 2 as Tabert et al. (2002) and Munro et al. (2018) did not define their specific criteria for Alzheimer's diagnosis despite only examining this dementia outcome.

### Measures of awareness

All 11 studies used a different measure when scoring awareness (see, Table 2). Four used patient-only measures, the remaining 7 incorporated an informant.

#### Patient-only measures

Wolfsgruber et al. (2014) asked participants “Do you feel like your memory has become worse?”: 1. “No”; 2. “Sometimes, but this does not worry me”; 3. “Yes, that worries me”; 4 “Yes, that worries me seriously”. Of interest for this review were the patients who responded 1. “No”, indicating a lack of awareness (*N* = 19, see Table 1). Cova et al. (2017) retrospectively analysed the response to the question: “Do you feel you have more problems with your memory than most?” from the Geriatric Depression Scale [47]. Patients were classified as ‘unaware’ if they responded “No”.

Nobili et al. (2010) administered the Memory Complaint Questionnaire (MAC-Q) in their study. This consists of 6 questions where respondents are asked to compare their current memory abilities to the past. Scores range from 7 (memory now is better than in the past) to 35 (memory now much worse than in the past). The authors used a cut-off score of ≤ 25 to indicate that a patient had impaired awareness of their current memory abilities—‘MCI/unaware’.

Finally, Munro et al. (2018) measured patients’ subjective memory complaints using the first 18 questions of the General Frequency of Forgetting subscale of the Memory Functioning Questionnaire (MFQ) [48]. Objective memory performance was assessed using a composite score derived from standard episodic memory tests e.g., Wechsler Logical Memory Assessment [49]. Awareness of memory at both timepoints was measured by modelling the relationship between objective performance (on episodic tasks) and subjective complaints (questions from the MFQ), with the residual score reflecting the degree to which self‑reports did not align with actual performance.

#### Patient/informant measures

Seven studies included an informant in their measure of awareness. Gifford et al. (2014) carried out interviews with patients and informants. Patients were divided into 4 groups: 1) no cognitive complaint 2) self-cognitive complaint only 3) informant cognitive complaint only and 4) both self and informant cognitive complaint. Of interest for the current review was group 3.

Five studies measured awareness by investigating the discrepancy between patient and informant scores on a questionnaire, all of these studies chose different questionnaires. Gerretsen et al. (2017) used the Everyday Cognition—Study Partner Report (ECog-PR) and Everyday Cognition – Participant Self-Report (ECog-SR) [46] asking participants to compare current abilities to 10 years ago. Silva et al. (2016) used the 16-item Forgetfulness Assessment Inventory (FAI) [21] assessing how often memory difficulties were encountered in the past 4 weeks. Tabert et al. (2002) used the Pfeffer Functional Activities Questionnaire (FAQ) [50] and the Lawton Independent Activities of Daily Living (IADL) Scale [51]. These questionnaires focus on patients’ awareness of their ability to complete day to day activities such as handling finances, housekeeping, remembering appointments and medication, and independently travelling. Spalletta et al. (2014) used the 19-item Memory Insight Questionnaire (MIQ) [52]; a 19-item questionnaire assessing the perception of cognitive and functional deficits. It consists of a total score and 4 sub-scores: general functioning, memory, language, and cognitive-general-executive. Chang et al. (2023) used the 17-item Consortium to Establish a Registry for Alzheimer’s Disease (CERAD) clinical history questionnaire [53] for which patients and informants were asked to give a yes/no/don’t know response about their performance across several different cognitive domains – such as word list learning and verbal fluency.

Finally, Bastin et al., (2021) collected patient and informant reports on the Memory Functioning Scale from the Memory Awareness Rating Scale (MARS-MFS) [54]. In addition, patients performed a feeling of knowing (FOK) experimental task for semantic and episodic memory. This was used as an objective measure of awareness, as participants were asked to metacognitively judge their ability to recognise a learned stimulus.

### Prevalence of unawareness of cognitive deficits in MCI

Four studies defined awareness categorically as either ‘aware’ or ‘unaware’. Estimated prevalence rates of unawareness varied considerably between the studies. Cova et al. (2017) defined 164 out of 282 aMCI patients as unaware, giving a prevalence rate of 58.2%. Nobili et al. (2010) defined 17 out of 42 aMCI patients, giving a prevalence rate of 40.5%. Gifford et al. (2014) described 151 MCI patients out of 1843 as unaware, suggesting only a prevalence of 8.2%. Finally, Wolfsgruber et al. (2014) found that only 19 MCI patients out of 417 denied any decline in memory, giving a prevalence of 4.6%.

### Overall measurement of rates of dementia conversion

The studies varied in whether they reported an overall conversion rate to dementia and whether they delineated different types of dementia. Chang et al. (2023) did not report an overall dementia conversion figure but stated that using Petersen’s MCI criteria, those with no self-reported cognitive complaints showed a 83.1% dementia conversion rate, compared to 44.4% in those with self-reported cognitive complaints. Cova et al. (2017) reported that 63.3% of their cohort developed dementia, of which the majority (68.8%) had Alzheimer’s Disease. Gifford et al. (2014) reported a conversion of 44% to “Alzheimer’s Disease, or other dementias”, but did not include a breakdown of conversion rates to different dementia subtypes. The remaining 8 studies investigated conversion to Alzheimer’s Disease only.

### Association between awareness and dementia conversion

Four studies did not find any significant association between awareness and dementia conversion [21, 34, 36, 38]. Six studies reported a significant association between awareness and dementia conversion [21–25, 33, 37]. One final study did not test statistical significance between awareness and dementia conversion [35].

#### Studies without a significant association between awareness and dementia conversion

Cova et al. (2017) did not find an impact of awareness on progression to dementia either when adjusting for confounding variables or without this adjustment HR = 0.838; 95% CI = 0.572–1.228, *p* > 0.05; HR = 0.991; 95% CI = 0.732–1.342, *p* > 0.05 respectively. Nobili et al. (2010) found that when adjusted for mean follow-up time, the annual conversion rate was statistically equivalent: 40.2% in ‘unaware’ group; 21.9% in ‘aware’ group. Nonetheless, all ‘unaware’ aMCI patients either remained as aMCI or developed AD at follow up, while 35.3% (6/17) of ‘aware’ aMCI patients reverted to normal cognition or their deficits were explained by depression, 9 remained stable, with the remaining 2 developing other forms of dementia. Silva et al. (2016) revealed a non-significant trend that awareness increased over time for stable MCI patients and decreased among those who converted to AD.

Chang et al. (2023) found that the MCI group most likely to develop dementia at follow-up were the group where cognitive complaints were endorsed by both the patient and the informant (HR = 5.14) followed by the ‘unaware’ group with high informant complaint but low self-report complaint (HR = 2.32). Having no cognitive complaints was associated with a higher conversion rate (83.1%) than low (74.2%) or high (44.4%) levels of complaints. Statistical significance was not investigated.

Wolfsgruber et al. (2014) similarly found that patients with no memory complaints or worry showed higher conversion rates (19/93, 31.6%), than those with little complaints and no worry (5/93, 5.4%), some worry (42/211, 19.9%), and serious worry (21/94, 22.3%). Statistical significance was not investigated.

#### Studies with a significant association between awareness and dementia conversion

The remaining 6 studies reported a significant association between awareness and dementia conversion. Munro et al. (2018) was the only study to find a significant association when patient self-report alone was involved in measuring awareness. This was the case when adjusting for confounding variables (age, sex, years of education, and follow up time: HR = 0.35, 95% CI = 0.143–0.857, *p* = 0.018).

Three studies comparing informant to patient responses using questionnaires to measure awareness found an association between unawareness and dementia. Spalletta et al. (2014) found that patient-informant discrepancy on the memory subscale of the MIQ significantly predicted dementia conversion (U = 63.000, z = 2.366, *p* = 0.0180). At baseline, larger discrepancies were also seen on language and general executive subscales among converters compared to non/converters but these were not significant.

Gerretsen et al. (2017) found that unawareness measured through composite ECog-SR and ECog-PR scores significantly predicted conversion from MCI to AD within 5 years, through logistic regression. This was with (OR = 1.64, 95% CI 1.12–2.5, *p* = 0.011) and without (OR = 2.74, 95% CI = 1.95–8.85, *p* < 0.001) confounding variables.

Tabert et al. (2002) also found that baseline informant-patient discrepancy significantly predicted AD conversion (F[1,87] = 6.6, *p* < 0.012). MCI patients who would later convert to AD had significantly greater informant reported deficits on the Pfeffer FAQ, whilst those who remained as MCI showed the reverse (ANCOVA rater x diagnostic outcome interaction: (F[1,87] = 6.6, *p* < 0.012). This suggests that converters may under-report their deficits, while non-converters may over-report them. A Cox proportional hazards model showed that having greater informant reported functional deficits than self-reported deficits on the Pfeffer FAQ was a significant risk factor for converting to AD (RR = 4.4, 95% CI = 1.8–10.6, *p* = 0.001) after controlling for age, MMSE score, and years of education. However, the same finding was not observed when patient and informant discrepancy on the Lawton IADL scale was used as the predictor (RR = 2.6, 95% CI = 0.9–7.2, *p* = 0.078). This suggests that a discrepancy between informant and self-report on some measures of functional abilities is predictive of conversion to AD at follow up.

Gifford et al. (2014) found that MCI patients who denied cognitive complaints but whose informants endorsed them, were more likely to convert to dementia than MCI patients where neither patient nor informant endorsed cognitive difficulties (OR = 2.2; 99%CI = 1.2–4.3, *p* < 0.001), when controlling for age, sex, race, education, follow-up period, and MMSE score at baseline.

Finally, Bastin et al. (2021) found converters to dementia had a significantly higher baseline discrepancy between informant and patients compared to non-converters and controls (F(2, 66) = 8.44, *p* < 0.001, η 2p = 0.20). On their episodic (but not semantic) FOK task, converters made fewer correct predictions about their ability to recognise a learned stimuli than controls (*p* < 0.05) but non-converters did not (*p* > 0.05). There were no significant correlations between any of the FOK performance measures and the discrepancy index on the MARS (*p*s > 0.35).

## Conclusions and discussion

This systematic review examined whether reduced awareness of cognitive deficits predicts conversion from MCI to dementia. Six studies found a significant association [21–25, 33, 37], while four found a non-significant or untested association [34–36, 38], and one found no link [21]. Overall, reduced awareness is associated with an increased dementia risk, though effect sizes varied, likely due to methodological differences. Higher-quality studies were more likely to report significant associations. Of the five high-quality studies, three found significant links, and two did not test significance. In contrast, the only study reporting no association [21] and one of the two non-significant studies were rated as low quality [34]. Generally, studies of higher quality were more likely to find a significant association compared to the weaker quality studies.

Studies comparing objective cognitive performance with subjective memory complaint score found that those who endorsed the fewest complaints exhibited the poorest objective performance [35] and were at higher risk of conversion to dementia [37]. Patients who would later convert to dementia also overestimated their performance on episodic (but not semantic) memory FOK tasks [25]. A high discrepancy between subjective complaints and objective cognitive performance seems to be associated with increased risk of dementia conversion, particularly in the episodic memory domain. This aligns with research suggesting that episodic memory ability is impacted more greatly by AD due to the degeneration in neural networks implicated in this domain [55].

Measuring the discrepancy between informant and patient reports appeared a more sensitive measure of dementia risk than patient self-reports alone. The exception was when the Lawton IADL was used, where no association was observed between discrepancy and dementia conversion [33]. A significant finding was observed in the same study using the Pfeffer FAQ however, suggesting the Lawton IADL may not be the best measure to capture awareness. A potential reason for this discrepancy might be that the Lawton IADL is much shorter and less detailed than the Pfeffer FAQ and so is less sensitive. Informants may also be helpful in differentiating between individuals with genuine cognitive impairment and those who 1) are ‘worried well’ or 2) have symptoms better explained by depression, anxiety, or other mental health difficulties. Hypervigilance of cognitive decline being associated with more symptoms of depression and anxiety compared to those with reduced awareness of their cognitive decline has been shown elsewhere [56]. Indeed, Nobili et al. (2010) found that at follow-up a proportion of their ‘aware’ cohort had deficits better explained by depression than MCI. None of the studies included in this review systematically investigated the relationship between awareness of cognitive and/or functional abilities and other neuropsychiatric symptoms such as anxiety, apathy, or disinhibition, which is an area for potential future exploration.

Due to our criteria, some papers relevant to the topic were not included. For example, papers using the same database as another study already included in the review—the Alzheimer’s Disease Neuroimaging Initiative (ADNI). Evidence from these related studies also supported the hypothesis that unawareness during MCI was associated with an increased risk of conversion to dementia [see 57–60].

Our findings have important implications for clinical practice; awareness should ideally be assessed during MCI diagnosis. While NICE guidance states that informants are recommended to be included in dementia consultations [61], the role of informants in MCI diagnoses is less clear [62]. The findings here suggest that a combination of informant report, patient report, and objective measures of cognitive function to identify a patient’s level of awareness are more effective than measures involving the patient alone. However, not every patient presenting to a memory clinic or with a diagnosis of MCI will have a reliable informant available. Chang et al. (2023) reported that only 62% of their overall sample could provide an informant, and this subset had an over-representation non-Hispanic White patients. Previous studies have suggested that older adults who are socially isolated (and therefore unlikely to have an informant) may in fact be at greater risk of developing dementia [63]. Therefore, while informants may add valuable insight for MCI patients, clinicians must be wary of disadvantaging health disparate groups. An additional point is that the reliability of informants might be impacted by many issues – such as their own burden through caregiving, the quality of the relationship with their loved one and their own mood. Indeed, there are alternative ways of measuring awareness in clinical settings. This could be done via obtaining a brief measure of self-appraisal during standard cognitive screening task such as the MoCA [64] and comparing this to objective performance. These methods could require little time and resources and will identify individuals who may be at greater risk of conversion to dementia. Research aimed at establishing consensus on the most appropriate measure of awareness to use during MCI diagnosis would be highly valuable. For instance, a Delphi approach could help facilitate expert agreement and reduce the current methodological heterogeneity, ensuring that discrepancies between measures do not impede scientific progress.

Relevant to this work, the National Institute on Aging and the Alzheimer’s Association (NIA AA) have refined the accepted criteria for diagnosing MCI over recent years [65]. Their guidelines address both diagnosis in the absence of biomarker evidence and research identifying biomarkers of early disease—prior to dementia onset—when advanced neuroimaging (e.g., amyloid PET imaging) and/or cerebrospinal fluid analysis is available [66, 67]. However, despite the promise of biomarkers that can indicate disease progression before clinical diagnosis of dementia, access to these diagnostic tools remains inequitable. This highlights the potential value of identifying simple, affordable behavioural indicators—such as an awareness check—that could help flag individuals who may benefit from further biomarker assessment. Given the emerging progression of biomarker evidence in this area, an important next step would be to conduct a systematic review or, if feasible, a meta-analysis to evaluate whether reduced awareness in individuals with MCI is consistently associated with specific biomarkers [59] In relation to this, the increased use of biomarkers to diagnose dementias might lead to better understanding of the incidence of a lack of awareness both pre-diagnosis of AD and after-diagnosis.

There are limitations to our review. First, the articles included are heterogenous, particularly in the way they measured and characterised awareness. This meant that it was not possible to conduct a meta-analysis. This limitation highlights an important implication. Of the 8 studies in the current review using questionnaires to measure awareness, all used a different questionnaire. The absence of a ‘gold standard’ measure, limits the confidence we can have in drawing conclusions more broadly. The variability in methodologies used extends to the variability of the statistical tests applied and the confounding variables included in these – this also limits this review from drawing stronger conclusions. Not all of the included papers accounted for a fully comprehensive set of potentially confounding variables. Examination of the confounders controlled for in Table 3, together with the CASP quality appraisal of how studies accounted for confounders (Table 4), suggests that higher-quality studies which identify and adjust for a more comprehensive set of confounders might be more likely to report an association between lack of awareness of impairment and subsequent dementia diagnosis. Of the three high-quality studies that accounted for a comprehensive range of confounders, two reported a statistically significant association between lack of awareness of impairment and conversion to Alzheimer’s disease at follow-up [23, 24]. The third did not formally test the significance of this association; however, its results indicate that a greater proportion of participants with lack of awareness of impairment at baseline were diagnosed with dementia at follow-up compared with those who did not show lack of awareness (83.1% vs 44.4%) [38]. These findings highlight an area that warrants further investigation in future research.

Second, 5 studies were identified through forward and backward citation searching rather than from the literature database searches. This suggests that our initial search terms were not broad enough to capture all relevant articles, despite being reviewed by all 3 authors and a specialist librarian. We are optimistic that the inclusion of forward and backward citations captured the remaining relevant papers, but the possibility remains that some studies were missed. Finally, only 5 of the 11 studies were classed as high quality. Common limitations included small sample sizes, short follow up duration, and failing to identify and/or account for all confounding factors in the design and analysis. We should therefore be wary of drawing conclusions based on some of the poorer quality studies, and further research is needed that addresses common limitations; this research should include consideration of potential differences between amnestic and non-amnestic MCI and standardise the follow-up period over which conversion is examined. As not all confounding factors are consistently considered across these papers we cannot yet say for certain that a lack of awareness is an *independent* risk factor for an eventual dementia diagnosis.

In addition, although the CASP tool is widely used to judge quality of research, its application inevitably involves a degree of subjective judgement when deriving an overall quality rating from the 12 individual criteria. This subjectivity is difficult to avoid given the diversity of study designs included in our review, but it remains an important limitation to acknowledge. A further potential limitation to consider is that measures of patient rated cognitive impairment might be affected by non-disclosure by the patient for reasons other than lack of awareness, for example some research has considered stigmatisation [68]. It should be noted that the current review cannot uncover the mechanisms through which awareness differences lead to an increased likelihood of a dementia diagnosis and understanding this a crucial step.

Finally, only two of the included studies assessed biomarkers associated with Alzheimer’s disease at follow-up, both of which used fluorine-18 fluorodeoxyglucose positron emission tomography (FDG-PET) [24, 34]. None of the studies used amyloid-PET to confirm dementia diagnoses, despite this being considered a current gold standard for identifying Alzheimer’s pathology. This limitation may affect the strength of the conclusions, as participants diagnosed with Alzheimer’s disease at follow-up may not all have had pure Alzheimer’s pathology.

An important direction for future research, and one which is not accounted for across the current papers, concerns the conceptual complexity surrounding awareness. For example, in the current literature, patients may be described as presenting with subjective memory difficulties, anosagnosia, or metacognitive deficits and although these constructs all reflect reduced awareness of impairment, they may arise from different underlying mechanisms. For example, mood disturbances such as anxiety may lead to distorted monitoring of cognitive performance, whereas impairments in awareness may also arise from disruptions to cognitive monitoring processes themselves, potentially linked to broader executive dysfunction.

A further challenge is that metacognition itself operates at multiple levels, which are rarely examined simultaneously. Many existing studies assess higher-level metacognitive beliefs using questionnaire measures, capturing general beliefs about one’s cognitive abilities. In contrast, relatively few clinical studies measure local metacognition, such as trial-by-trial confidence and its calibration with performance, or global metacognition, which reflects confidence about overall task performance. Evidence suggests that these levels may dissociate in clinical populations [69], raising the possibility that different profiles of metacognitive impairment exist across individuals. In some cases, impairment across multiple levels of metacognition may reflect a more pervasive disruption of self-monitoring and could potentially signal a higher risk of progression to Alzheimer’s disease. In fact, differences across levels of metacognitive awareness have been documented in Alzheimer’s disease [70], highlighting the relevance of assessing these processes earlier in the disease trajectory. Relatedly, the importance uncovered here concerning discrepancies between self-report and informant report likely reflect underlying metacognitive deficits rather than simply differences in perspective. Conceptualising such discrepancies within formal models of metacognitive monitoring, similar to those used in experimental paradigms [71] may provide a more precise way of characterising impaired awareness. Characterising how different aspects of metacognition change across stages of cognitive decline may therefore help clarify the mechanisms underlying reduced awareness and improve identification of individuals at greater risk of progression. For a broader theoretical framework on self-awareness in dementia and other neuropsychiatric conditions, see [72].

In conclusion, poor awareness of cognitive decline is a likely risk factor for MCI-to-dementia conversion. Based on the papers assessed in our review, this association appears to be stronger when awareness is assessed using informant input and/or objective comparisons, rather than patient self-report alone. Clinicians should attempt to assess awareness during MCI diagnosis as patients with reduced awareness may benefit from closer monitoring. Future research may involve 1) investigating the relationship between reduced awareness to other neuropsychiatric conditions, 2) working towards a ‘gold standard’ measure of awareness, particularly regarding the vast heterogeneity in questionnaires currently in use, and 3) adding to the current literature with high quality studies using detailed, lengthy follow-ups and larger sample sizes.

## Supplementary Information

Supplementary Material 1.

## Data Availability

No datasets were generated or analysed during the current study.

## Abbreviations

AD: Alzheimer’s disease
ADNI: Alzheimer’s Disease Neuroimaging Initiative
CASP: Critical Appraisal Skills Programme
CINAHL: Cumulative Index to Nursing and Allied Health Literature
MCI: Mild Cognitive Impairment
MoCA: Montreal Cognitive Assessment
NIA AA: National Institute on Aging and the Alzheimer’s Association
NICE: National Institute for Health and Care Excellence.
PRISMA: Preferred Reporting Items for Systematic Reviews and Meta Analyses
PROSPERO: Prospective Register of Systematic Reviews
