# Supplementary material for: Unawareness of deficits in mild cognitive impairment: a systematic review of its role in progression to Alzheimer’s disease
Source: BMC Neurol. 2026 May 20;26:396. doi: 10.1186/s12883-026-04884-8 (PMC13281629; doi:10.1186/s12883-026-04884-8)
Supplement: Supplementary file 1 — Supplementary Material 1. [file 12883_2026_4884_MOESM1_ESM.docx]

# **Supplementary Information**

## **Full Search Strategy:**

**Embase**

| **Framework** | **Subject headings** | **Free text searching** |
| --- | --- | --- |
| Population | Mild cognitive impairment | Cognit* adj4 impair* OR MCI OR cognit* adj4 decline OR cognit* adj4 dysfunction* OR cognit* adj4 difficult* OR memory adj4 decline OR predementia OR pre-clinical adj4 dementia OR prodromal adj4 dementia OR cognit* adj4 deteriorat* |
| Intervention/exposure | Introspection OR awareness OR metacognitive awareness OR anosognosia OR metacognition OR metamemory OR self-evaluation | Introspect* OR insight OR awareness OR anosognosia OR metacognit* OR metamemory OR appraisal OR evaluation |
| Comparison | N/A | N/A |
| Outcome | Prediction OR early diagnosis  AND  Dementia OR Alzheimer disease OR multiinfarct dementia OR diffuse Lewy body disease OR frontotemporal dementia OR senile dementia OR presenile dementia | Predict* OR progress* OR early adj4 detect* OR early adj4 diagnos* OR convert OR conversion OR transition  AND  Dementia OR Alzheimer* OR Lewy OR neurodegen* |
| Study design | Longitudinal study OR follow up OR prospective study | 1 Clinical study/  2 Case control study  3 Family study/  4 Longitudinal study/  5 Retrospective study/  6 Prospective study/  7 Randomized controlled trials/  8 6 not 7  9 Cohort analysis/  10 (Cohort adj (study or studies)).mp.  11 (Case control adj (study or studies)).tw.  12 (follow up adj (study or studies)).tw.  13 (observational adj (study or studies)).tw.  14 (epidemiologic$ adj (study or studies)).tw.  15 (cross sectional adj (study or studies)).tw.  16 Or/1-5,8-15 |

**Medline**

| **Framework** | **Subject headings** | **Free text searching** |
| --- | --- | --- |
| Population | Cognitive dysfunction OR cognition disorders | Cognit* adj4 impair* OR MCI OR cognit* adj4 decline OR cognit* adj4 dysfunction* OR cognit* adj4 difficult* OR memory adj4 decline OR predementia OR pre-clinical adj4 dementia OR prodromal dementia OR cognit* adj4 deteriorat* |
| Intervention/exposure | Awareness OR metacognition | Introspect* OR insight OR awareness OR anosognosia OR metacognit* OR metamemory OR appraisal OR evaluation |
| Comparison | N/A | N/A |
| Outcome | Early diagnosis  AND  Dementia OR frontotemporal dementia OR vascular dementia OR dementia, multi-infarct OR Alzheimer disease OR Lewy body disease OR neurodegenerative diseases | Predict* OR progress* OR early adj4 detect* OR early adj4 diagnos* OR convert OR conversion OR transition  AND  Dementia OR Alzheimer* OR Lewy OR neurodegen* |
| Study design | Longitudinal studies OR cohort studies OR follow-up studies OR prospective studies | 1 Epidemiologic studies/  2 Exp case control studies/  3 Exp cohort studies/  4 Case control.tw.  5 (cohort adj (study or studies)).tw.  6 Cohort analy$.tw.  7 (Follow up adj (study or studies)).tw.  8 (observational adj (study or studies)).tw.  9 Longitudinal.tw.  10 Retrospective.tw.  11 Cross sectional.tw.  12 Cross-sectional studies/  13 Or/1-12 |

**PsychINFO**

| **Framework** | **Subject headings** | **Free text searching** |
| --- | --- | --- |
| Population | Mild cognitive impairment OR cognitive impairment | Cognit* adj4 impair* OR MCI OR cognit* adj4 decline OR cognit* adj4 dysfunction* OR cognit* adj4 difficult* OR memory adj4 decline OR predementia OR pre-clinical adj4 dementia OR prodromal dementia OR cognit* adj4 deteriorat* |
| Intervention/exposure | Insight OR awareness OR metacognition OR anosognosia OR self-evaluation | Introspect* OR insight OR awareness OR anosognosia OR metacognit* OR metamemory OR appraisal OR evaluation |
| Comparison | N/A | N/A |
| Outcome | Prediction OR disease progression  AND  Dementia OR dementia with Lewy bodies OR semantic dementia OR senile dementia OR presenile dementia OR vascular dementia OR Alzheimer’s disease OR neurodegenerative diseases | Predict* OR progress* OR early adj4 detect* OR early adj4 diagnos* OR convert OR conversion OR transition  AND  Dementia OR Alzheimer* OR Lewy OR neurodegen* |
| Study design | Longitudinal studies OR prospective studies | Longitudinal stud* OR cohort stud* OR follow-up stud* OR prospective stud* OR population-based stud* |

**CINAHL**

| **Framework** | **Subject headings** | **Free text searching** |
| --- | --- | --- |
| Population | Mild cognitive impairment | Cognitive impairment OR cognitive decline OR cognitive difficulties OR cognitive dysfunction OR cognitive deterioration OR memory decline |
| Intervention/exposure | Self-awareness OR anosognosia OR introspection OR self-assessment | Awareness OR anosognosia OR introspection OR insight OR metacognition OR metamemory OR self-assessment OR appraisal OR evaluation |
| Comparison | N/A | N/A |
| Outcome | Early diagnosis OR disease progression  AND  Dementia OR frontotemporal dementia OR dementia, vascular OR dementia, multi infarct OR dementia, senile OR dementia, presenile OR lewy body disease OR neurodegenerative diseases | Early diagnosis OR progression OR predict* OR transition* OR convert* OR conversion  AND  Dementia OR alzheimer* OR Lewy OR neurodegen* |
| Study design | Longitudinal studies OR prospective studies | 1 Prospective studies/  2 Exp case control studies/  3 Correlational studies/  4 Nonconcurrent prospective studies/  5 Cross sectional studies/  6 (cohort adj (study or studies)).tw.  7 (observational adj (study or studies)).tw.  8 or/1-7 |
